# An end-to-end detection and classification model for tea leaf grading in complex orchard environments

**DOI:** 10.3389/fpls.2026.1814763

**Published:** 2026-06-05

**Authors:** Wencheng Hong, Tao Wang, Weiwei Zu, Miao Huan, Lingyi Liang, Liangquan Jia, Chong Yao

**Affiliations:** 1 School of Information Engineering, Huzhou University, Huzhou, China; 2 Huzhou Central Hospital, Affiliated Central Hospital of Huzhou University, Huzhou, China

**Keywords:** feature enhancement, lightweight network, object detection, open-field tea garden, RT-DETR, tea grade determination

## Abstract

**Introduction:**

Existing detection models for automatic tea-grade determination in open-field, complex habitats suffer from insufficient feature robustness, weak suppression of background interference, and difficulty in balancing lightweight design with accuracy. To address these limitations, this study proposes Tea-DETR, a model tailored for open-air tea garden scenarios based on the end-to-end RT-DETR detection paradigm.

**Methods:**

The proposed method enhances feature representation and attention allocation from two aspects: backbone optimization and feature interaction enhancement. First, a lightweight backbone module is designed to strengthen long-range dependency modeling and improve suppression of complex background interference, thereby enhancing the robustness and discriminability of multi-scale tea-leaf features under occlusion and illumination variations. Second, an efficient attention-enhancement mechanism is introduced to reduce redundant feature interactions and enable adaptive focus on critical target regions, improving the model’s ability to capture subtle semantic differences among tea grades while maintaining computational efficiency. To validate the approach, Anji white tea from Zhejiang Province was selected as the experimental subject, and a single-leaf tea dataset containing 6,542 images was constructed. The dataset was split into training, validation, and test sets at a ratio of 8:1:1, based on which comparative experiments and convergence analyses were conducted.

**Results:**

With the number of parameters reduced to 14.65M, Tea-DETR achieves an accuracy of 92.2% and improves mAP@0.5 to 82.0%, reducing parameters by 26.8% compared with the baseline model. In addition, Tea-DETR exhibits markedly improved convergence stability and substantially enhanced capability in distinguishing subtle semantic differences among tea grades, effectively alleviating ambiguous discrimination under complex backgrounds.

**Discussion:**

Overall, the proposed method enhances the stability of fine-grained feature capture for small tea-leaf targets in open-field environments while maintaining real-time inference efficiency, providing a robust solution for real-time, non-destructive automatic tea-grade determination in field scenarios.

## 1 Introduction

Tea, as one of the most widely consumed functional beverages worldwide, has a quality grade thatnot only directly determines its market value but also influences consumers’ sensory experience and health safety (Abbasi et al., 2025). Within modernized tea production and precision agriculture systems, accurate tea-grade determination serves as a critical basis for processing decisions, quality traceability, and price setting (Altemimi et al.,). Traditionally, appearance attributes—such as tenderness, color, and strip integrity—are regarded as key phenotypic cues for grade differentiation. However, conventional tea classification still relies heavily on manual labor, resulting in low efficiency and strong variability due to assessors’ expertise and environmental conditions (Bakhshipour et al., 2018). Therefore, it is of particular importance to develop techniques capable of timely and accurate tea-grade inspection.

With rapid advances in optoelectronics and artificial intelligence, computer vision (CV) has become a mainstream approach for non-destructive inspection of agricultural products. Early studies on tea largely employed conventional machine-learning methods, in which shallow features (e.g., color moments and texture descriptors) were manually designed and extracted for classification or detection (Chen et al., 2021; Cao et al., 2025). Chen et al. (2025) proposed a tea-quality evaluation method based on near-infrared spectroscopy combined with a random forest classifier, achieving efficient and low-cost quality classification. Adel Bakhshipour et al (Chen et al., 2024). extracted color and texture features from images and optimized an artificial neural network (ANN) using correlation-based feature selection, obtaining a classification accuracy of 96.25% for four black tea varieties. Yan et al (Ding et al., 2024). performed key-feature selection via random forest recursive feature elimination (RF-RFE) and constructed a tea classification model using SVM. Nevertheless, such handcrafted features exhibit limited generalization in complex, unstructured environments and are often incapable of handling illumination variations and diverse leaf poses (Feng et al., 2021).

In recent years, the emergence of convolutional neural networks (CNNs) has substantiallyalleviated this bottleneck. Deep learning models represented by the YOLO family and Faster R-CNN,benefiting from strong feature self-learning capability, have achieved remarkable performance in crop disease diagnosis, fruit counting, and maturity assessment, and have also provided new technical routes for automated tea grading (Frontiers | WMC-RTDETR: a lightweight tea disease detection model; Girshick, 2015). Ding et al (Hassan et al., 2022). proposed a tea-grade classification method based on hyperspectral imaging spatial information and CNNs, where a ResNet-50 model significantly improved grading performance. Wang et al (He et al.,). introduced a Transformer module and coordinate attention into YOLOv7 to achieve accurate tea-bud grade detection under complex backgrounds. Zhu et al (Hu et al., 2021). built a ResNet50-based model for fast and accurate classification of five oolong tea varieties, reaching an accuracy above 93%. Xu et al (Lee et al.,). proposed a fusion network that combines the fast detection capability of YOLOv3 with the high-accuracy classification capability of DenseNet201, effectively addressing the recognition challenge of tea buds with similar colors in complex scenes. Considering the resource constraints of embedded devices, Tang et al (Lipiński et al., 2025). developed a lightweight tea-bud grading detector based on an improved YOLOv5, achieving a favorable trade-off between performance and efficiency. Despite these advances, most CNN-based detection studies are conducted in controlled settings, and their deployment in open-field, complex habitats remains challenging (Mangla et al., 2022).

With the introduction of Transformer architectures into vision tasks, end-to-end object detectionparadigms have exhibited superior potential for global modeling compared with conventional CNN-based pipelines (Mohyuddin et al., 2024). Chen et al (Saleem et al., 2019). developed a non-destructive fresh-leaf inspection system by combining a portable spectrometer with an improved Transformer architecture, achieving high accuracy in cultivar and quality classification of Wuyi rock tea. Zhan et al (Shi,). proposed IterationViT, a tea-disease recognition model that integrates CNNs with an iterative Transformer, reaching 98% classification accuracy by jointly exploiting local and global representations. Zhang et al (Tang et al., 2025). proposed WMC-RTDETR, a lightweight model improved from RT-DETR, achieving 97.7% detection accuracy and effectively addressing occlusion-related tea-disease detection in complex backgrounds.

However, although RT-DETR (Tang et al., 2023) has demonstrated excellent real-time performance and accuracy in general object detection, its application to fine-grained tea-grade determination remains at an early stage. When directly transferred to open-field tea gardens with complex habitats, two common bottlenecks persist. First, during backbone feature extraction, illumination fluctuations, background texture interference, and overlapping occlusions by branches and leaves can easily induce representation drift, causing fine-grained cues highly relevant to grade discrimination to be diluted through deep propagation or dominated by background noise. Second, during high-level semantic interaction, global attention mechanisms in complex scenes may trigger ineffective information exchange and impose additional computational burden, thereby weakening the model’s focus on key target regions and its cross-environment generalization.

To fill this gap and overcome the above limitations, this work systematically optimizes the RT-DETR end-to-end detection paradigm and proposes Tea-DETR, a model designed for open-field complex habitats:

1. Backbone optimization: We design a unified lightweight backbone module, C2f-EViM-CGLU. Built on the multi-branch aggregation structure of C2f, the module integrates EfficientViMBlock to enhance efficient long-range dependency modeling and introduces a ConvGLU context-aware gating mechanism to dynamically suppress interference from weeds, occlusions by mature leaves, and illumination-induced pseudo-responses, thereby producing multi-scale feature representations with improved robustness and purity.
2. Feature enhancement: We propose an AIFI_EPGO_SHSA module. While preserving the overall topology where AIFI operates only on high-level features, SHSA is incorporated to reduce attention redundancy and memory overhead, and EPGO is employed to achieve input-adaptive dynamic Top-k sparsification. This strategy concentrates a limited attention budget on critical tokens highly correlated with target leaves, substantially reducing ineffective feature interactions under complex backgrounds.

With these improvements, Tea-DETR maintains the advantage of real-time inference while significantly enhancing the stability of capturing fine-grained textures and boundary cues of small tea-leaf targets in open-field conditions, thereby providing a more robust solution for real-time, non-destructive automatic tea-grade determination in field environments.

The remainder of this paper is organized as follows. Section 2 describes the materials and methods, including dataset collection, the overall network architecture, and the proposed model components. Section 3 presents the experimental design and analysis, including dataset splitting, parameter settings, convergence analysis, ablation studies, comparative experiments, and experimental results. Section 4 discusses the findings and performance of the proposed method. Finally, Section 5 summarizes the conclusions of this study and provides directions for future research.

## 2 Materials and methods

### 2.1 Dataset collection

In this study, Anji white tea (Camellia sinensis cv. ‘Anji Baicha’) from the core production area of Anji County, Huzhou City, Zhejiang Province, China, was selected as the experimental subject. The region features a unique hilly microclimate and dense vegetation cover, forming a typical unstructured agricultural habitat. Image acquisition was conducted in open-field tea plantations to capture the morphological evolution of tea shoots under natural growth conditions.

**Figure 1:**
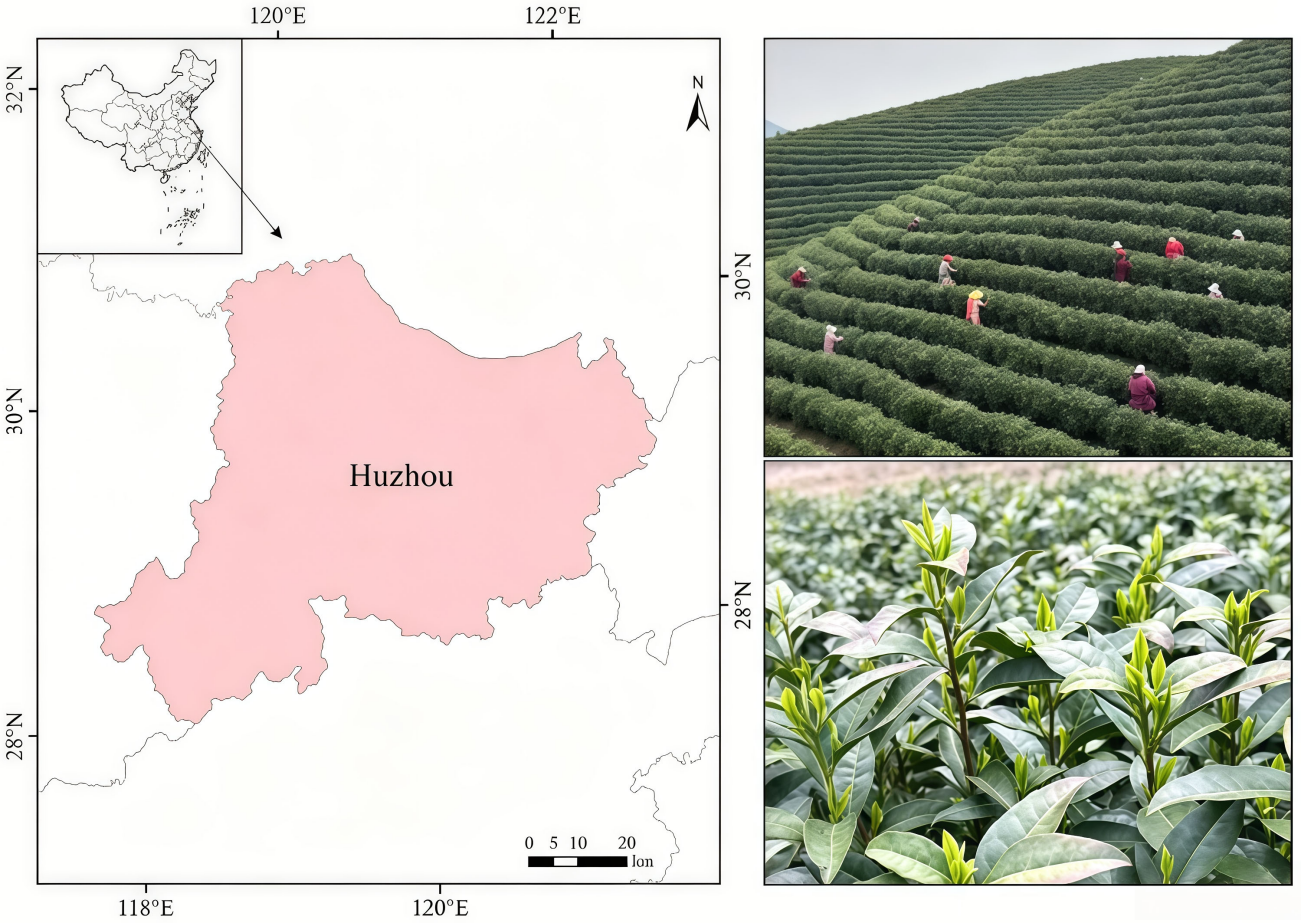
Field photograph of the tea-leaf data collection area.

A mobile imaging device with high photosensitivity was used for data collection. All images were recorded at a resolution of 4032 × 3024 pixels and stored in JPEG format. To accurately capture the fine-scale texture and color gradation of tea buds, the vertical distance between the camera lens and the tea canopy was strictly maintained within 15–25 cm during shooting. In addition, autofocus compensation was employed to effectively mitigate motion blur caused by ambient wind, ensuring clear delineation of bud contours and leaf edges. **Figure 1** illustrates the real ecological habitat of the experimental site.

To mitigate the interference of drastic illumination fluctuations in open-field environments on color-feature extraction for single leaves, sampling was conducted throughout the entire phenological period of spring tea harvesting. Data acquisition covered diverse natural weather conditions, including heavy morning fog, intense midday sunlight, and shaded afternoon scenes, thereby effectively capturing the dynamic physiological process of individual leaves from initial albinism to gradual greening. Considering the complex field conditions such as leaf overlap and background weed interference, a multi-dimensional image repository was established, including near-vertical top-down views, oblique side views, and frontal views at the canopy level. This long-term, multi-view acquisition strategy provided RT-DETR with sufficient unstructured environmental cues, substantially improving its detection and classification performance for both small targets (tender leaves) and large targets (mature leaves) in complex scenarios.

**Figure 2:**
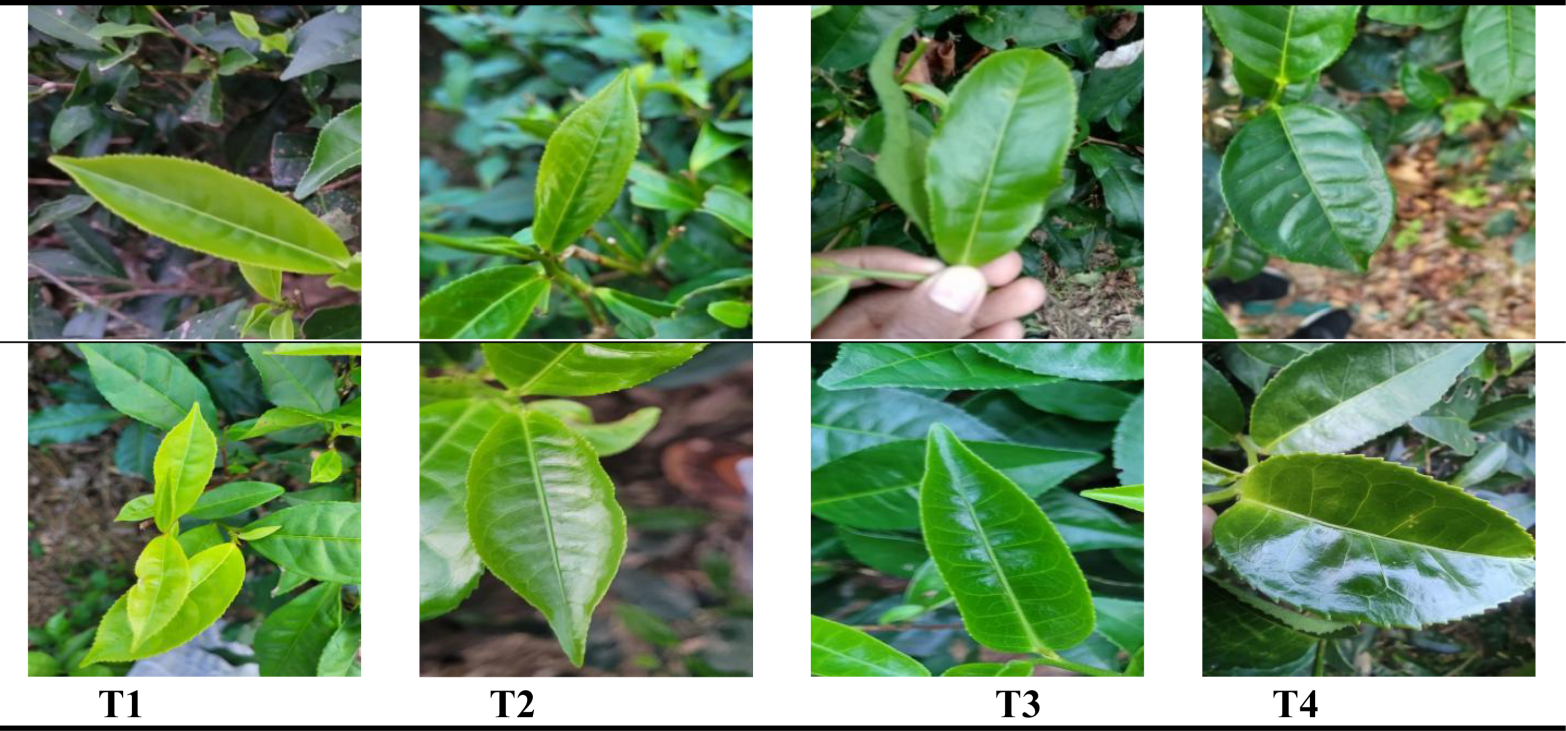
Example images of different tea-leaf grades.

In this study, single tea leaves were categorized into four grades, T1–T4 (from high to low), according to developmental stage, tenderness, and visual characteristics. The grading criteria are summarized as follows. T1 (ultra-tender leaf) refers to newly emerged young leaves that are extremely small, typically semi-curled or partially unfolded, with very delicate texture and a distinct jade-white or pale pinkish-green appearance, representing the most vigorous part of the plant. T2 (tender leaf) corresponds to largely expanded single leaves; compared with T1, they exhibit moderate leaf area with incipiently visible veins, and their color transitions between jade-white and bright green while retaining high physiological tenderness. T3 (early mature leaf) denotes fully flattened leaves with noticeably increased geometric size, clear serrated margins, uniformly green coloration, and a texture that begins to shift from soft to tougher. T4 (fully mature leaf) represents completely matured large leaves; these leaves reach maximal area and appear dark green with a glossy surface, accompanied by increased thickness and a high degree of lignification, and they often serve as prominent background or lower-layer leaves in images. Although the T1–T4 classification proposed in this study is not a standardized tea grading system in the traditional sense, it is closely related to actual quality assessment and harvesting requirements in tea production. Given that developmental stage, tenderness, and morphological appearance are key indicators influencing tea quality and harvesting value, this paper uses the term “grade” to describe a fine-grained classification framework for individual leaf identification in natural field environments. **Figure 2** illustrates representative detection outputs of the proposed model for single-leaf grading: red bounding boxes precisely localize highly tender T1 leaves, which are mainly distributed at the top of the tea canopy, whereas orange bounding boxes identify large-area, dark-colored T4 mature leaves.

### 2.2 Overall network architecture

To address the challenges of drastic scale variation of individual tea leaves and highly overlapping background textures in unstructured tea-garden habitats, this study proposes an end-to-end real-time detection model termed Tea-DETR. The proposed model is designed to overcome the inference stability and efficiency bottlenecks of conventional YOLO-family detectors in complex scenes, which largely stem from their reliance on non-maximum suppression (NMS) as a post-processing step (Wang et al., 2023). In end-to-end detection, the task is reformulated as a direct mapping from raw pixels to a set of predictions, where global context modeling is leveraged to eliminate the interference introduced by manually engineered components. Tea-DETR follows and further optimizes the DETR (Detection Transformer) paradigm (Xu et al., 2022). Unlike traditional detectors that perform predictions over dense candidate regions, DETR introduces bipartite matching and a fixed number of object queries; the Transformer decoder models inter-object relations via self-attention and directly generates a unique, non-redundant set of predictions, enabling a clean end-to-end inference pipeline without NMS-induced degradation.

The overall topology of Tea-DETR consists of a lightweight backbone, an improved efficientTransformer encoder, and a real-time end-to-end decoder, aiming at real-time detection and grade determination of single-leaf targets in natural tea-garden scenes. For the backbone, constrained by the multi-scale interface of RT-DETR (i.e., outputting {S3, S4, S5} for subsequent encoding), we propose c2f_EfficientVIM_ConvGLU (C2f-EVC) to replace ResNet (Yang et al., 2025). The backbone adopts the partial aggregation structure of C2f as its skeleton, and introduces the state-space feature mixing unit of EfficientViM (Yashodha and Shalini, 2021) only within the “re-branching” of partial channels, thereby enabling long-range dependency modeling and significantly enlarging the effective receptive field during feature extraction while keeping computational cost manageable. Meanwhile, the channel mixer is replaced with ConvGLU (Yun and Ro,) (a convolution-gated GLU), which performs token-level dynamic gating to suppress spurious responses induced by mature leaves, weeds, and background textures, improving feature purity and discriminability under strong illumination/shadow variations and occlusion overlap in open-field conditions.

In the feature-enhancement stage, we further propose AIFI_SHSA_EPGO. This module preserves RT-DETR’s design principle of conducting same-scale interaction only on the highest-level semantic features, but replaces the attention computation in AIFI with SHSA (Single-Head Self-Attention) (Zhan et al., 2023), where single-head self-attention is applied to only a subset of channels and combined with full-channel output projection, thereby reducing multi-head redundancy and memory-access overhead. On this basis, we introduce an EPGO-guided dynamic Top-k sparsification strategy: the sparsity level is adaptively determined by an input-conditioned prompt, forcing attention selection to concentrate on key tokens relevant to target leaves. This design reduces erroneous interactions with irrelevant regions in complex backgrounds and strengthens the modeling of fine-grained texture and boundary cues for small targets, particularly ultra-tender leaves (T1).

**Figure 3:**
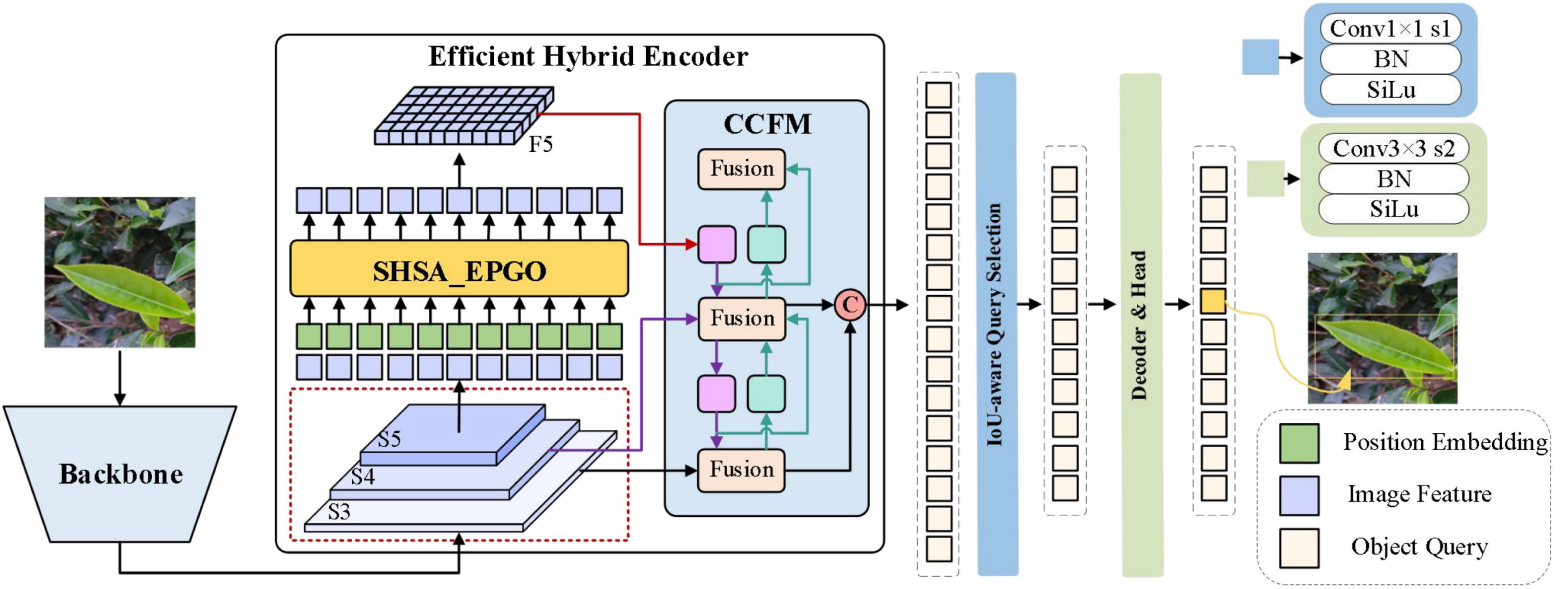
Overall network architecture.

During decoding, Tea-DETR follows the RT-DETR end-to-end decoding paradigm and directly outputs the geometric bounding boxes and grade categories (T1–T4) of individual tea leaves without NMS post-processing, providing a deployable solution for real-time and stable tea-grade determination in tea-garden scenarios. The overall network architecture is shown in **Figure 3**.

#### 2.2.1 Tea-DETR backbone

As a classic convolutional neural network architecture, ResNet18 has demonstrated strong performance across a wide range of computer vision tasks; however, it exhibits notable limitations when confronted with the complex backgrounds commonly observed in tea-garden environments. Tea-garden images are typically characterized by severe illumination fluctuations, weed interference, frequent leaf occlusions, and large target scale variations, which together pose substantial challenges to backbone feature extraction.

**Figure 4:**
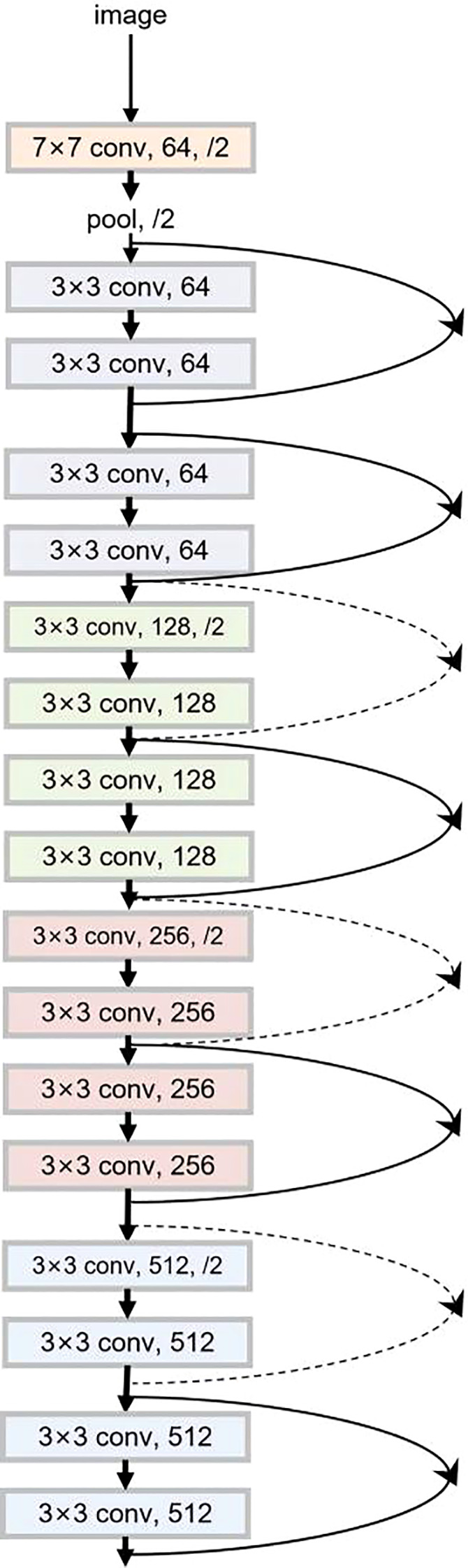
ResNet18 backbone.

Specifically, although ResNet18 can effectively capture local patterns, its receptive field expands relatively slowly with increasing convolutional depth. Meanwhile, the stacked design leads to a rapid increase in computational cost and parameter count as the network deepens. Consequently, ResNet18 may fail to sufficiently model global context when handling multi-scale targets and cluttered backgrounds. In addition, ResNet18 is less robust to background noise and occlusions, which can induce unstable feature representations and ultimately degrade the accuracy of subsequent detection and tea-grade determination. The backbone architecture of ResNet18 is illustrated in **Figure 4**.

**Figure 5:**
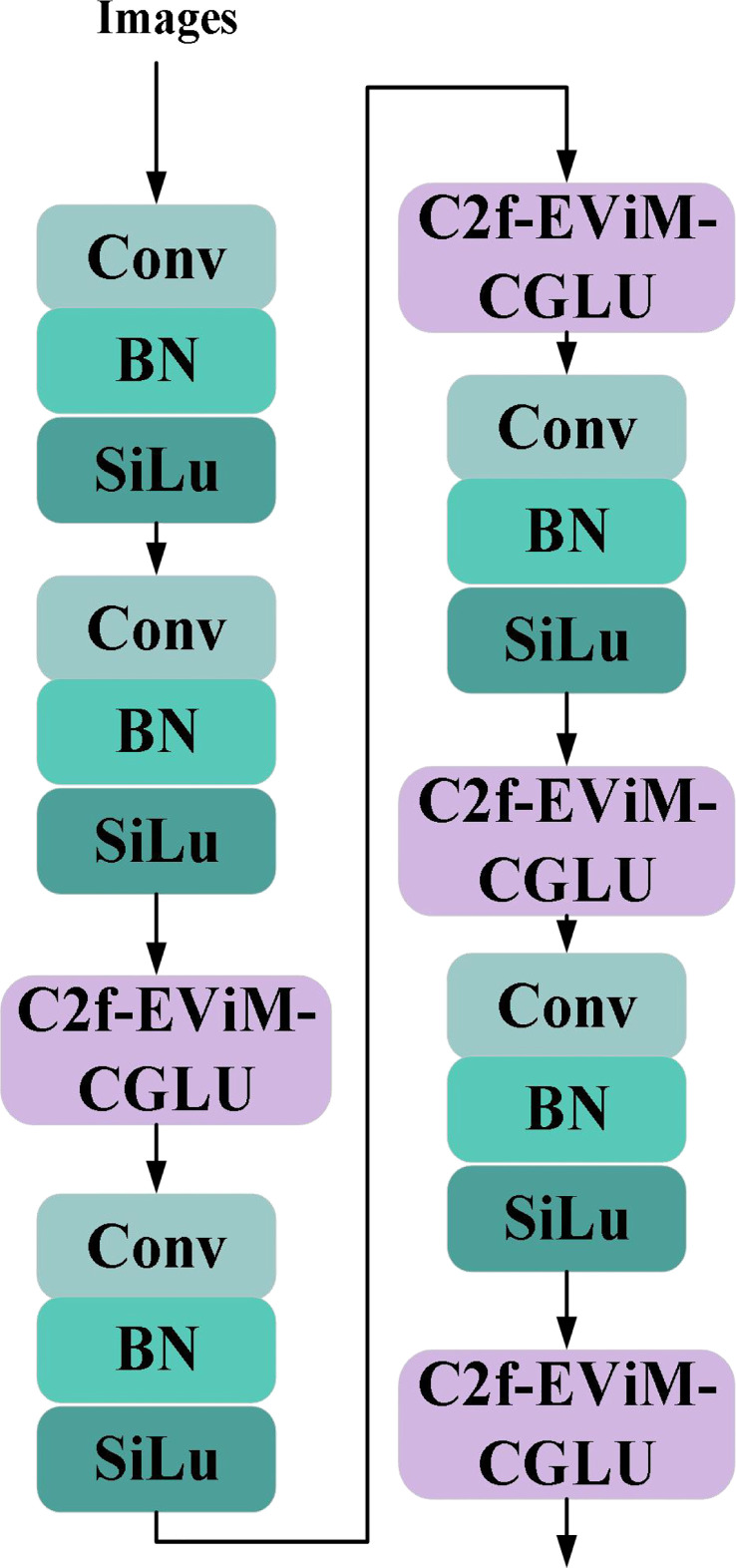
Tea-DETR backbone architecture.

To overcome the limitations of ResNet18 in tea-garden image analysis, this study proposes a novel backbone design. The new backbone not only retains the strengths of convolutional neural networks, but also incorporates feature fusion and enhancement mechanisms to enable stable extraction of high-quality multi-scale representations under complex backgrounds. The key innovation lies in integrating the C2f-EViM-CGLU module, which effectively fuses local and global information during feature extraction and strengthens global modeling capability. The architecture of the Tea-DETR backbone is shown in **Figure 5**.

The proposed backbone performs progressive downsampling through convolutional operations to effectively reduce the spatial resolution of feature maps while gradually increasing the number of channels, thereby capturing hierarchical image information at multiple levels. After each convolutional stage, a subsequent C2f-EViM-CGLU module further refines the features by suppressing background noise and enhancing discriminative details. Through adaptive attenuation of redundant responses caused by weeds and occlusions, these modules emphasize weak-contrast textures of tender leaves, fine leaf-edge structures, and key cues related to color and surface characteristics, ultimately improving the accuracy of tenderness-grade determination.

#### 2.2.2 C2f_EfficientVIM_ConvGLU

Tea-garden images captured under natural field conditions often contain severe illumination variation, weed interference, leaf occlusion, and multi-scale targets, which place higher demands on feature robustness and global contextual modeling. Conventional convolutional backbones mainly rely on local receptive fields and deep stacking, making them prone to redundant background activations and unstable feature representations in cluttered tea-garden environments. These limitations are particularly unfavorable for fine-grained discrimination of weak-contrast tender leaves such as T1/T2 categories.

To address these issues, we propose a lightweight backbone module, C2f-EViM-CGLU, within the YOLO-style C2f framework. The proposed design aims to jointly enhance long-range dependency modeling and adaptive background-noise suppression while maintaining efficient computation. Specifically, EfficientViM is introduced to strengthen global contextual interaction, whereas ConvGLU dynamically filters irrelevant activations caused by weeds, shadows, and mature-leaf occlusions, thereby improving the discriminability of fine-grained tea-leaf features.

**Figure 6:**
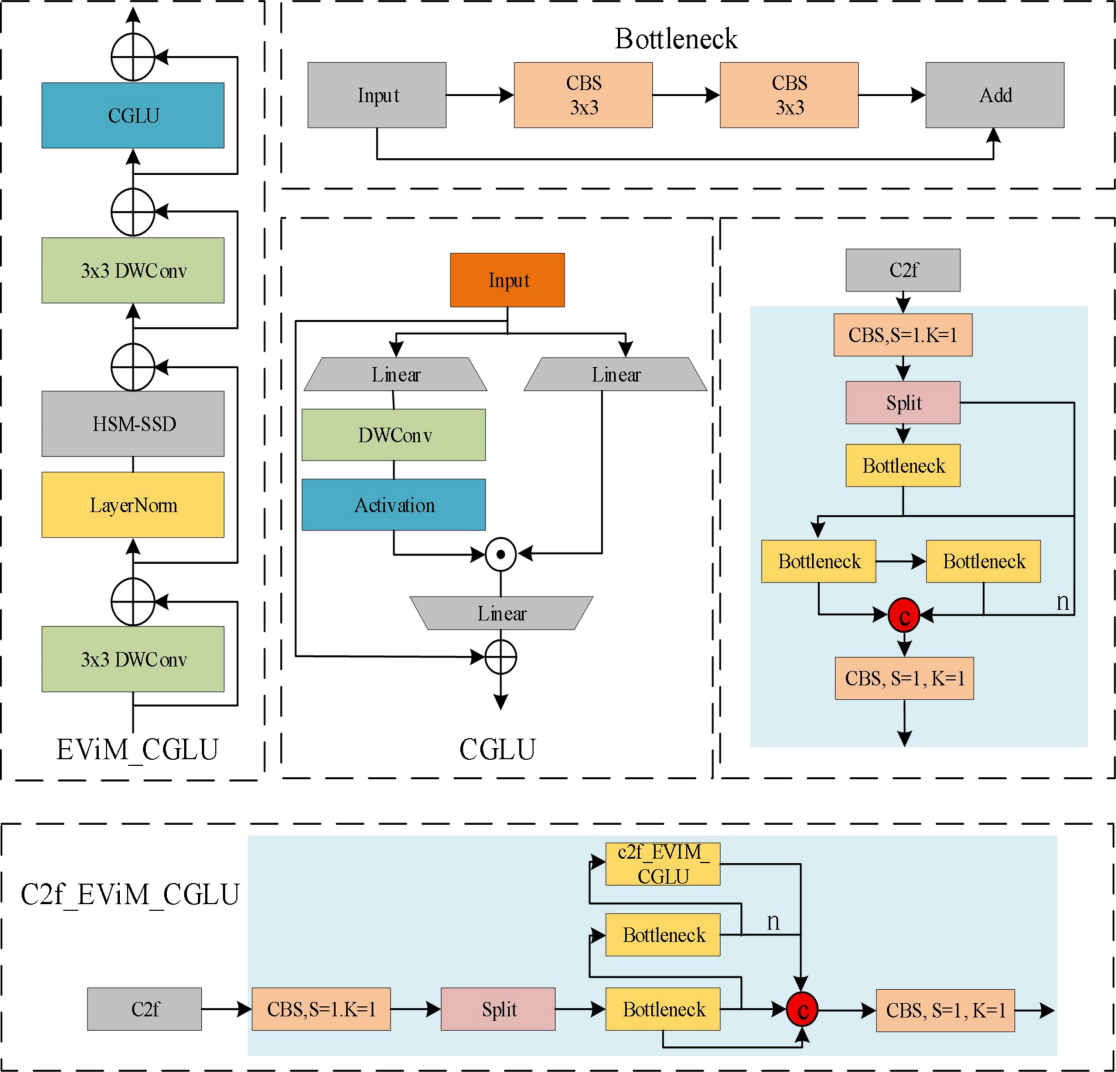
Architecture of the C2f-EViM-CGLU module.

With these designs, the backbone yields multi-scale feature representations that are cleaner, more focused, and more stable, while remaining friendly to real-time inference. This provides a solid foundation for efficient feature interaction in the subsequent encoder and for end-to-end grade prediction. The structure of the C2f-EViM-CGLU module is illustrated in **Figure 6**.

In practice, the proposed design is realized through two complementary components.

(1) Design I: Efficient global mixing via EfficientViM under the C2f framework

We adopt the YOLO-style C2f architecture featuring partial-channel computation and multi-branch aggregation, such that relatively heavy representation enhancement is applied only to a subset of channels. This strategy improves representational capacity while maintaining high throughput. Let the input feature map be $\text{X}\in {\mathbb{R}}^{\text{B}\times \text{C}\times \text{H}\times \text{W}}$. We first perform channel alignment and split it into a light branch and a heavy branch as shown in Equation 1:

(1) $$U={\mathrm{Conv}}_{1\times 1}\left(X\right),\left({\text{U}}_{0},{\text{U}}_{1}\right)=Split\left(\text{U}\right)$$

where U_0_ denotes the light branch that bypasses computation to preserve gradient flow and control cost, and U_1_ is fed into the heavy branch. The heavy branch stacks n enhancement units (the proposed EVC block, defined later) as formulated in Equation 2:

(2) $${\text{Z}}_{0}={\text{U}}_{1},\;{\text{Z}}_{\text{i}}={\text{B}}_{\text{EVC}}\left({\text{Z}}_{\text{i}-1}\right),\text{i}=1,\ldots \ldots \text{n}$$

Finally, multi-branch features are aggregated and fused to produce the output as shown in Equation 3:

(3) $$\text{Y}={\text{Conv}}_{1\times 1}\left(\mathrm{Concat}\left({\text{U}}_{0},{\text{U}}_{01},{\text{Z}}_{1},\ldots \ldots {\text{Z}}_{\text{n}}\right)\right)$$

Within this C2f skeleton, to obtain more stable long-range dependency representations, we introduce into B_EVC_ the idea of hidden-state-driven global mixing: spatial tokens are first mapped into a smaller set of hidden states, global interaction is performed in the hidden-state space, and the result is then reconstructed back to the original token space. For simplicity, we flatten the input into a token sequence as shown in Equation 4:

(4) $$\text{x}=\text{Flatten}\left(\text{X}\right)\in {\text{R}}^{\text{B}\times \text{L}\times \text{D}},\text{ L}=\text{HW}$$

The global mixer consists of three steps—compression, mixing, and reconstruction as formulated in Equations 5–7:

(5) $$\text{h}=\;\Phi \left(\text{x}\right)\in {\text{R}}^{\text{B}\times \text{N}\times \text{D}},\text{ N}\leq \text{L}$$

(6) $$\bar{\text{h}}=\text{S}\left(\text{h}\right)$$

(7) $$\text{GlobalMix}\left(\text{x}\right)=\Psi \left(\bar{\text{h}}\right)\in {\text{R}}^{\text{B}\times \text{L}\times \text{D}}$$

where Φ(·) and Ψ(·) are learnable compression/reconstruction mappings, and S(·) is a mixing operator applied in the hidden-state space. Intuitively, global information propagation is carried by a much smaller hidden-state set of size N, avoiding explicit L×L pairwise interactions and thus keeping computation and memory growth more manageable for high-resolution features.

This design improves global contextual representation and enhances robustness against occlusion and scale variation.

(2) Design II: Noise suppression and enhancement via context-aware gating (ConvGLU)

Global mixing alone does not automatically eliminate open-field background noise. Weed textures, shadow highlights, and large dark regions contributed by mature leaves can produce strong yet grade-irrelevant activations in deep semantic features. To address this, we incorporate ConvGLU into the same enhancement unit B_EVC_ as a dynamic gating modulator. Specifically, local context is leveraged to generate gating weights that perform element-wise filtering on content features, thereby suppressing redundant responses and strengthening fine-grained cues relevant to grading.

ConvGLU can be expressed concisely as shown in Equation 8:

(8) ConvGLU(X)=ϕ(X)⊙g(DWConv(ψ(X)))

Where *ϕ*(·) is the content-mapping branch, ψ(·) is the gating branch (typically implemented by 1×1 convolutions. DWConv(·) injects local contextual information, g(·) is the gating activation function, and ⊙ denotes element-wise multiplication.

By combining the above two mechanisms, the proposed backbone core unit is defined as shown in Equation 9:

(9) $${\text{B}}_{\text{EVC}}\left(\text{X}\right)=\text{X}+\text{Reshape}\left(\text{GlobalMix}\left(\text{Flatten}\left(\text{DWConv}\left(\text{X}\right)\right)\right)\right)+\text{ConvGLU}$$

This formulation highlights the key properties of our backbone: (i) DWConv preserves convolutional inductive bias for local texture, preventing global mixing from weakening edge details; (ii) GlobalMix (hidden-state mixing) provides stable long-range dependency modeling; and (iii) ConvGLU adaptively suppresses unstructured background noise while enhancing grade-discriminative features.

Finally, the backbone outputs multi-scale features as shown in Equation 10:

(10) $$\left\{\text{S}3,\text{S}4,\text{S}5\right\}={\text{Backbone}}_{\text{EVC}}\left(\text{I}\right)$$

which are directly aligned with the input interface of the subsequent encoder, enabling seamless replacement of the conventional backbone without altering the overall topology of the detection head.

#### 2.2.3 AIFI_EPGO_SHSA

AIFI is an important intra-scale interaction module in the RT-DETR hybrid encoder, designed to enhance global semantic interaction on high-level feature maps. In Tea-DETR, the high-level semantic feature S5 is used for intra-scale interaction to improve fine-grained tea-grade recognition and bounding-box localization under complex tea-garden environments.

However, conventional multi-head self-attention (MHSA) still suffers from two limitations in open-field tea-garden scenarios: excessive computational redundancy and ineffective interactions caused by background noise tokens. Complex regions such as weeds, mature leaves, and illumination artifacts may introduce large numbers of irrelevant tokens, thereby weakening the model’s attention to subtle structural details of tender leaves.

**Figure 7:**
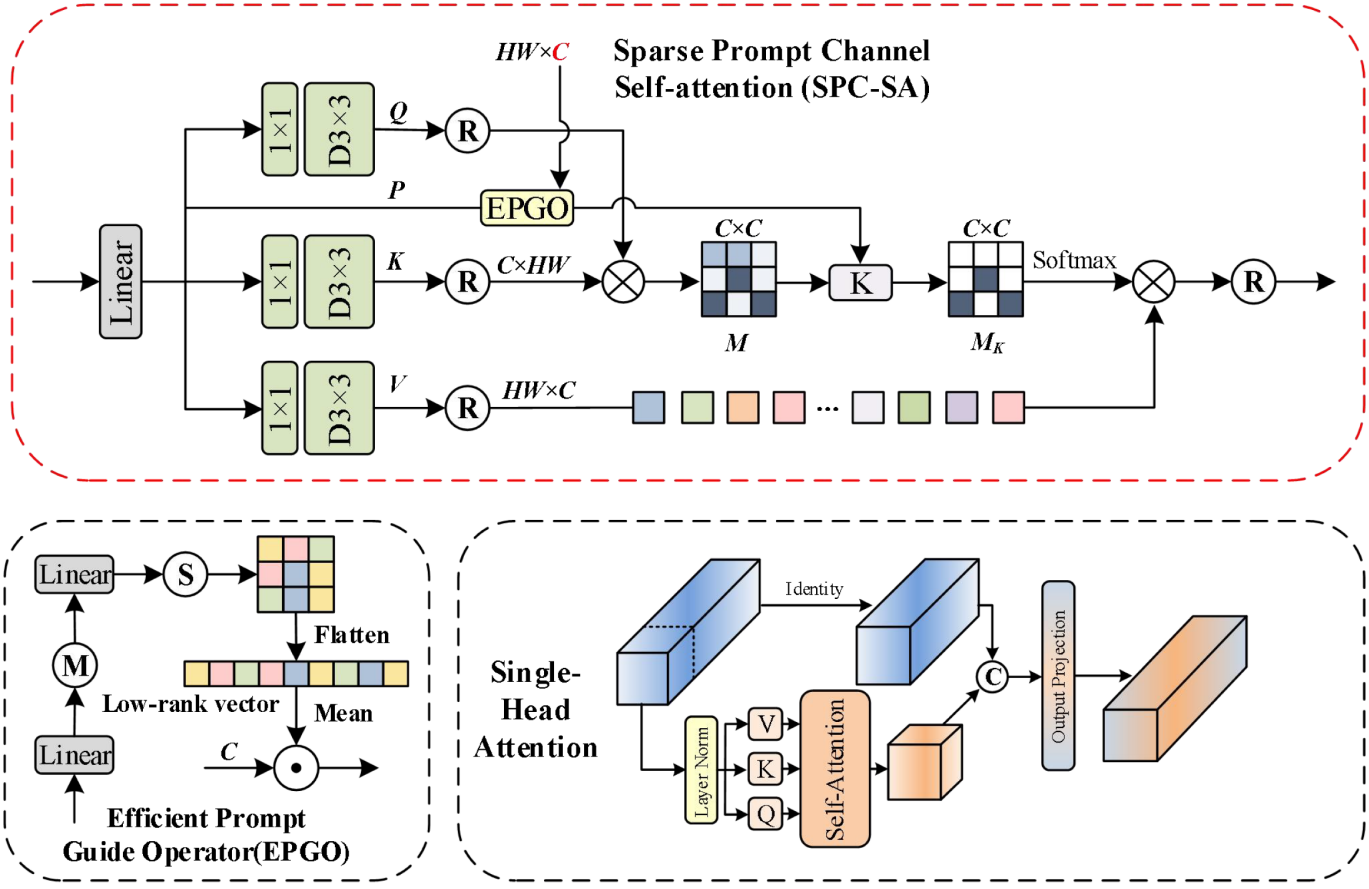
Architecture of the AIFI_EPGO_SHSA module.

To address these issues, we propose AIFI_EPGO_SHSA. Without altering the overall topology of AIFI—i.e., operating only on high-level semantic features—we introduce Single-Head Self-Attention (SHSA) proposed in SHViT (Zhan et al., 2023) as the intra-scale interaction operator. By applying single-head attention to only a subset of channels, SHSA effectively reduces head redundancy and memory-access overhead inherent to MHSA. In addition, inspired by the Efficient Prompt Guide Operator (EPGO) in CPRAformer (Zhang et al., 2025), we employ a lightweight prompt-guided dynamic Top-k selection strategy to achieve input-adaptive attention sparsification. This mechanism allocates the attention budget more selectively to key tokens that are highly relevant to the target leaves under complex open-field backgrounds, thereby significantly improving robust representation learning in unstructured habitats and enhancing the capture of fine-grained details for small targets. The structure of the AIFI_EPGO_SHSA module is shown in **Figure 7**.

In the implementation, given the high-level feature map $\text{S}5\in {\mathbb{R}}^{\text{B}\times \text{C}\times {\text{H}}_{5}\times {\text{W}}_{5}}$, we first flatten it into a token sequence as shown in Equation 11:

(11) $$\text{X}=\text{Flatten}\left(\text{S}5\right)\in {\text{R}}^{\text{B}\times \text{L}\times \text{C}},\text{ L}={\text{H}}_{5}{\text{W}}_{5}$$

The core idea of SHSA is to apply single-head self-attention only to a subset of channels while keeping the remaining channels as a bypass, thereby reducing redundant attention computation and alleviating memory-access pressure. Specifically, we split the channels with a ratio r as shown in Equation 12:

(12) $$\left({\text{X}}_{\text{a}}{\text{,X}}_{\text{r}}\right)={\text{Split}}_{\text{C}}\left(\text{X};\text{rC},\left(1-\text{r}\right)\text{C}\right)$$

Single-head Q, K, and V are constructed from Xa as shown in Equation 13:

(13) $$\text{Q}={\text{X}}_{\text{a}}{\text{W}}_{\text{Q}},\text{ K}={\text{X}}_{\text{a}}{\text{W}}_{\text{K}},\text{ V}={\text{X}}_{\text{a}}{\text{W}}_{\text{V}}$$

and the attention logits are computed as shown in Equation 14:

(14) $$\text{A}=\frac{{\text{QK}}^{\text{T}}}{\sqrt{\text{d}}}$$

Notably, the advantage of SHSA does not lie in shrinking the interaction range; instead, it reduces multi-head redundancy via a single head and partial-channel attention while still preserving global interaction capability.

To mitigate ineffective interactions induced by background noise in open-field tea gardens, we further introduce EPGO-guided dynamic sparsification on the attention logits. Concretely, a prompt variable p is adaptively generated from the current input and mapped to a dynamic Top-k retention budget, so that attention is concentrated on more relevant tokens.

We generate a lightweight prompt as shown in Equation 15:

(15) p=σ(f(Pool(X_a))), p∈(0,1)

where Pool(·) denotes global pooling and f(·) is a lightweight mapping function. The dynamic Top-k budget is then defined as shown in Equation 16:

(16) $$\text{k}=\left[{\text{k}}_{\min}+\text{p}\cdot \left({\text{k}}_{\max}-{\text{k}}_{\min}\right)\right],\text{ k}\in \left[{\text{k}}_{\min},{\text{k}}_{\max}\right]$$

For each query, we retain only the Top-k positions in the logits and obtain the sparse attention output as shown in Equation 17:

(17) $${\hat{\text{X}}}_{\text{a}}=\mathrm{Softmax}\left(\text{A}⊕{\text{M}}_{\text{k}}\right)\text{V}$$

This mechanism allows the model to automatically adjust interaction density according to image complexity: when the background is more cluttered, occlusion is stronger, or the target is smaller and harder, attention tends to focus on a small set of the most relevant tokens; conversely, under cleaner backgrounds and clearer structures, richer contextual interactions are permitted, thereby balancing robustness and expressiveness.

Finally, the sparse-attention channels are concatenated with the bypass channels, and an output projection is applied to propagate attention information to all channels, followed by reshaping to restore the spatial structure as shown in Equation 18:

(18) $$\text{Y}=\mathrm{Concat}\left({\hat{\text{X}}}_{\text{a}},{\text{X}}_{\text{r}}\right){\text{W}}_{\text{O}},\;{\text{F}}_{5}=Reshape\left(\text{Y}\right)\in {\text{R}}^{\text{B}\times \text{C}\times {\text{H}}_{5}\times {\text{W}}_{5}}$$

The resulting F5, together with {S3,S4}, is then fed into subsequent cross-scale fusion, keeping the overall Tea-DETR encoder topology unchanged and forming a consistent enhancement pipeline from local modeling to global interaction and finally to cross-scale aggregation.

In tea-garden environments, tender leaves such as T1/T2 categories often exhibit weak contrast and subtle texture differences, making them vulnerable to interference from weeds, mature leaves, and illumination artifacts. The proposed SHSA and EPGO mechanisms jointly improve the stability and effectiveness of high-level semantic interaction by reducing redundant attention computation and suppressing irrelevant token interactions. Consequently, the proposed module enhances fine-grained feature discrimination and provides more robust semantic representations for end-to-end tea-grade prediction.

## 3 Experiments and analysis

### 3.1 Dataset split

To evaluate the generalization capability and robustness of RT-DETR for tea-leaf grade classification, we constructed a high-quality single-leaf dataset consisting of 6,542 raw images. Prior to training, a stratified random sampling strategy was applied to balance the data, ensuring consistent grade distributions (T1–T4) across different subsets. Following standard experimental protocols in deep learning, the dataset was strictly divided into the training set, validation set, and test set at a ratio of 8:1:1.

The detailed split is as follows: the training set contains 5,234 images and is used for gradient-based weight updates and representation learning; the validation set contains 654 images and is used for hyperparameter tuning during training as well as monitoring potential overfitting; and the test set contains 654 images, which are excluded from any training or optimization and are used solely to assess the final recognition accuracy of single leaves across the four grades (T1–T4) under real, complex field conditions. This rigorous partitioning protocol provides a faithful reflection of the classification performance of lightweight models in unstructured backgrounds.

### 3.2 Experimental platform and parameter settings

To ensure efficient and robust training of RT-DETR, we established a dual-platform experimental setup following a “cloud training–local validation” workflow. Large-scale model training was conducted on an Ubuntu 22.04 server cluster, with computational acceleration provided by an NVIDIA A100 (80GB) Tensor Core GPU. Inference benchmarking and validation were performed locally on a workstation equipped with an Intel(R) Core(TM) i5-14600K CPU and an NVIDIA GeForce RTX 3090 GPU with 24GB VRAM.

On the software side, all experiments were implemented in PyTorch, with operator-level acceleration enabled by CUDA 12.4 and cuDNN. The input image size was unified to 640 × 640. Considering the convergence characteristics of the Transformer components in RT-DETR, we did not adopt conventional momentum-based SGD; instead, we used the AdamW optimizer with a weight decay coefficient set to 0.0001. Training was performed for 200 epochs, and a batch size of 32 was used to better utilize GPU throughput. The detailed environmental configuration and model hyperparameters are provided in **Table 1** and **Table 2**, respectively.

**Table 1:** Environment configuration.

Configuration	Local configuration	Server configuration
CPU	Intel Core i3 12100	Intel Core i9-14900hx
GPU	GeForce RTX 3090-24G	NVIDIA A100 Tensor Core GPU
Memory	64GB	128 GB
Operating system	Win 11	Ubuntu 22.04
python	3.8	3.8
pytorch	1.8	1.8
CUDA	11.2	11.2
cudnn	8.2	8.2

**Table 2:** Training hyperparameter settings.

Hyperparameter	Value
Initial learning rate	1×10-4
Optimizer	AdamW
Momentum	0.937
Weight Decay	1×10-4
Batch Size	32
Dropout Rate	0.1
epoch	200

### 3.3 Convergence analysis

**Figure 8:**
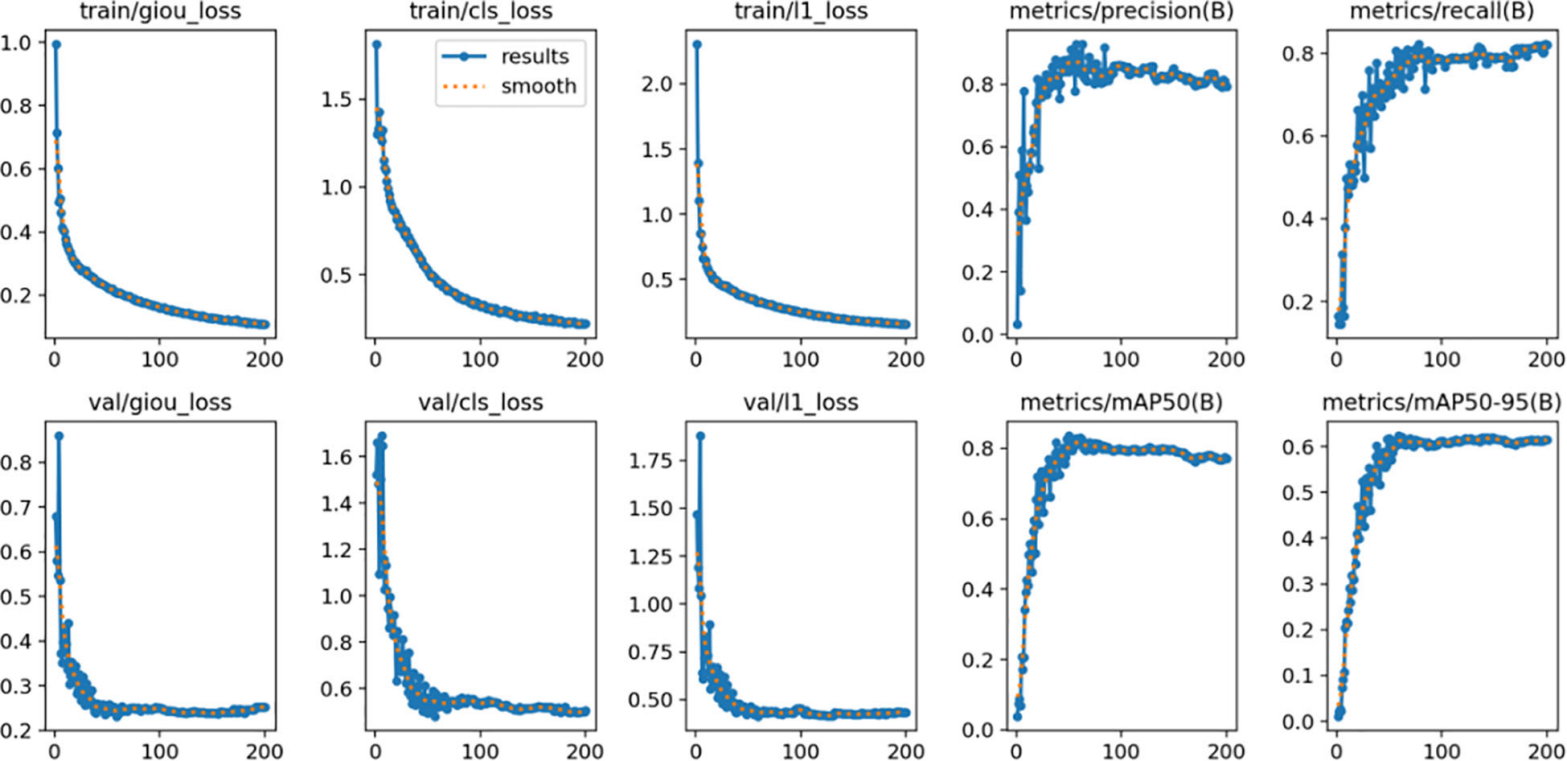
Curves of model performance metrics.

To evaluate the convergence behavior of the proposed model, we visualized the evolution trends of the loss functions and evaluation metrics during training. As shown in **Figure 8**, the losses train/giou_loss, train/cls_loss, and train/l1_loss decrease rapidly in the early training stage, and then the decreasing rate gradually slows down and becomes stable. Meanwhile, the validation losses val/giou_loss, val/cls_loss, and val/l1_loss exhibit a consistent downward trend with the training phase and enter a plateau in the mid-to-late stage. These results indicate that the optimization process is stable throughout training, without loss oscillation or divergence, suggesting that the model can effectively learn both localization and classification representations and achieves satisfactory overall convergence.

Further inspection of the detection metrics in **Figure 8** shows that Precision, Recall, mAP@0.5, and mAP@0.5:0.95 increase markedly during the early training stage and then gradually stabilize, indicating that the model’s detection capability on the validation set improves steadily as training proceeds and reaches a relatively stable level in the mid-to-late stage. Meanwhile, the performance gains in the late stage are limited, and some metrics exhibit slight fluctuations, suggesting that further increasing the number of training epochs would yield marginal improvements and that the model is already close to its optimal state. Overall, the loss and metric curves demonstrate that the trained model achieves good convergence and stability, meeting the requirements for subsequent experiments and practical deployment. In practice, it is recommended to select the checkpoint that attains the best validation performance as the final model to obtain more robust generalization.

### 3.4 Ablation study

To comprehensively quantify the contributions of the proposed improvements to the RT-DETR-R18 architecture for single-leaf tea grade classification, we conducted a series of ablation experiments using a decoupled verification strategy. The original RT-DETR-R18 was used as the baseline (Experiment A). We then incrementally integrated the proposed modules, including C2f-EfficientVIM (CEV) (Experiment B), the CGLU gating mechanism (Experiment C), and the AIFI-SHSA-EPGO (ASE) feature enhancement strategy (Experiment D), aiming to investigate the trade-off between computational cost and recognition accuracy. The evaluation metrics include model size (Parameters), computational complexity (GFLOPs), and mean average precision (mAP). Quantitative results of the ablation study are summarized in **Table 3**.

**Table 3:** Results of the ablation study.

Code	CEV	CGLU	ASE	Parameter/M	Precision/%	Recall/%	mAP@0.5/%	FLOPs/G
A				20.18M	83.8	77.4	81.7	56.8
B	✓			14.77	90.1	79.6	82	49.6
C	✓	✓		14.7M	91.7	80.1	82.3	49.6
D	✓	✓	✓	14.65M	92.2	79	83.2	48

The experimental results indicate that each proposed module provides clear benefits in both computational efficiency, feature robustness, and fine-grained discriminability under complex tea-garden environments. First, introducing C2f-EfficientViM (CEV) enables long-range dependency modeling with (approximately) linear complexity, alleviating the computational redundancy of convolutional layers when handling large-scale global backgrounds. Compared with the baseline, Scheme B reduces the parameter count by 26.8% while improving mAP@0.5 to 82.0%, demonstrating that this design enhances multi-scale feature representation and improves robustness against background texture interference and partial occlusion.

Second, the CGLU gating mechanism yields stronger discriminative power for single-leaf representations. Built upon CEV, Scheme C integrates a gated linear unit and further improves Precision to 91.7% without incurring additional computational overhead. Since single-leaf detection in natural tea gardens is frequently affected by overlapping leaves, weed interference, and illumination variations, CGLU dynamically suppresses irrelevant background responses while preserving critical edge and texture information of tender leaves. This enhancement contributes to more stable localization and improved discrimination among visually similar tea grades.

Finally, by integrating the AIFI-SHSA-EPGO (ASE) enhancement strategy, Scheme D achieves the best overall performance. Under an extreme lightweight setting with only 14.65M parameters, it attains a Precision of 92.2%. This result suggests that ASE, through global feature re-weighting and interaction-path optimization, effectively strengthens adaptive attention allocation to discriminative leaf structures while reducing redundant feature interactions. Consequently, the proposed strategy substantially improves robustness and fine-grained classification accuracy in complex open-field tea-garden habitats.

### 3.5 Comparative experiments

**Figure 9:**
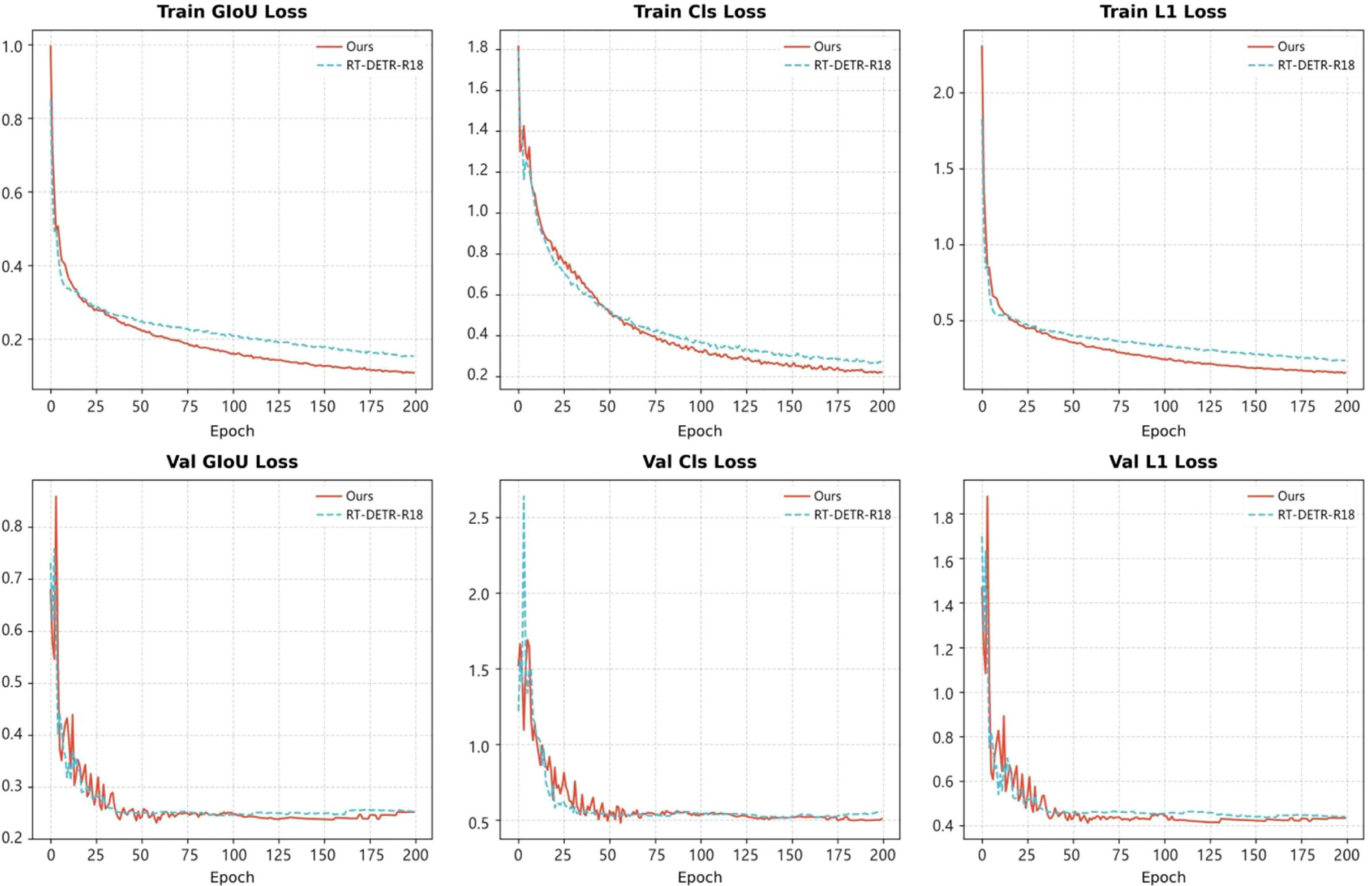
Training loss curves of RT-DETR-R18 and tea-DETR.

To further validate the learning effectiveness and convergence characteristics of the proposed Tea-DETR model for tea-grade determination, we compared the loss evolution of the baseline RT-DETR-R18 and the improved Tea-DETR over 200 epochs. As shown in **Figure 9**, three core losses were analyzed: GIoU Loss (localization loss), Classification Loss (classification loss), and L1 Loss (regression loss), assessed from both training and validation perspectives to reflect model fitting quality.

Overall, Tea-DETR exhibits superior convergence stability. For L1 Loss, the improved and baseline models show similar behavior, both achieving stable coordinate regression. For GIoU Loss, Tea-DETR demonstrates a slight but consistent advantage over RT-DETR, indicating that the improved backbone provides stronger geometric robustness in capturing precise boundaries of individual tea leaves.

The most significant improvement is observed in Classification Loss. The curves show that Tea-DETR reduces validation classification loss more rapidly and converges to a lower final value. This directly confirms that incorporating C2f-EfficientViM and the CGLU gating mechanism fundamentally strengthens the model’s ability to distinguish subtle semantic differences among tea grades (T1–T4), effectively alleviating the ambiguity that the baseline model tends to exhibit under complex backgrounds.

To further investigate the overall performance of the proposed improved model (Our) on thetea-leaf grade detection task, we conducted a comparative study against several representativemainstream object detection algorithms, including RT-DETR, Faster R-CNN (Zhao et al.,), TOOD (Zhu et al., 2021), RTMDet (Zhu et al., 2024), and Deformable-DETR (Zou et al., 2025). To ensure the fairness of cross-model comparisons, all compared models were trained and evaluated under identical experimental conditions, including the dataset split, input image resolution (640 × 640), optimizer configuration (AdamW), batch size (16), training epochs (200), and hardware platform. In addition, consistent data preprocessing and data augmentation strategies were applied across all experiments to avoid performance bias caused by differences in training configurations. The evaluation focused on four key aspects: model size (Parameters), Recall, detection accuracy (mAP@0.5), and computational cost (GFLOPs). The comparative results are reported in **Table 4**.

**Table 4:** Comparative experiments.

Model	parameter/M	recall/%	mAP@0.5/%	FLOPs/G	FPS
RT-DETR_R18	20.18	77.4	81.7	56.8	85.0
Faster RCNN_R50	28.3	68.8	82.4	120.5	30.4
TOOD_R50	32	61.1	74.1	95.8	31.3
RTMDet_tiny	8.5	73	79.8	65.4	91.8
Deformable DETR	44	75	81.1	90	56.1
Our	14.65	79	83.2	48	131.6

Based on the results in **Table 4**, to comprehensively assess the practical utility of the proposed Tea-DETR model for tea-grade classification, we benchmarked it against several representative object detection methods, including the two-stage detector Faster R-CNN and efficient one-stage approaches TOOD, RTMDet, and Deformable-DETR. The experimental results are summarized in **Table 5**. The data indicate that our model demonstrates a clear advantage in balancing accuracy and efficiency. In terms of detection accuracy, the proposed model (Ours) achieves an mAP@0.5 of 83.2%, outperforming both the baseline RT-DETR-R18 (81.7%) and the high-accuracy Faster R-CNN (82.4%). Meanwhile, it attains the highest Recall among all compared methods (79%), effectively reducing missed detections of tea-leaf targets in complex field backgrounds.

**Table 5:** Class-wise mAP@0.5 comparison across tea-leaf grades.

Classification	RT-DETR mAP@0.5/%	Ours mAP@0.5/%
T1	76.5	79.2
T2	80.8	82.5
T3	83.4	84.6
T4	86.1	86.5

From the perspective of lightweight deployment, our model reduces the parameter count to 14.65M, representing an approximate 27.4% reduction relative to the baseline, while requiring only 48 GFLOPs, the lowest computational cost among methods with comparable performance. In contrast, Deformable-DETR, which has the largest parameter count (44M), still yields lower accuracy (81.1%) than our approach. Although RTMDet-tiny is extremely compact, its 79.8% accuracy is insufficient for high-precision grade determination. These results demonstrate that reconstructing the backbone with C2f-EfficientViM and integrating the AIFI-SHSA-EPGO enhancement strategy substantially strengthens single-leaf feature extraction while markedly reducing hardware requirements, thereby meeting the real-time grading demands of mobile devices in unstructured tea-garden environments. At the same time, the experimental results in **Table 4** show that Tea-DETR maintains high detection accuracy while still delivering excellent real-time inference performance, further validating its potential for application in real-time tea leaf detection scenarios in the field.

According to the class-wise analysis in **Table 5**, T1 leaves are the most challenging category for all models due to their extremely small size and severe background interference. Nevertheless, benefiting from multi-scale feature enhancement enabled by C2f-EfficientViM and the ASE mechanism, Tea-DETR improves the T1 detection accuracy from 76.5% to 79.2%. As leaf maturity increases (T2–T4), visual cues become more salient and the detection accuracy rises steadily. Overall, the proposed model not only maintains a high recognition rate for large T4 mature leaves, but also significantly strengthens the learning and detection capability for high-value T1 tender buds, confirming its superiority in unstructured tea-garden habitats.

### 3.6 Analysis of detection and Grad-CAM++ visualization results

**Figure 10:**
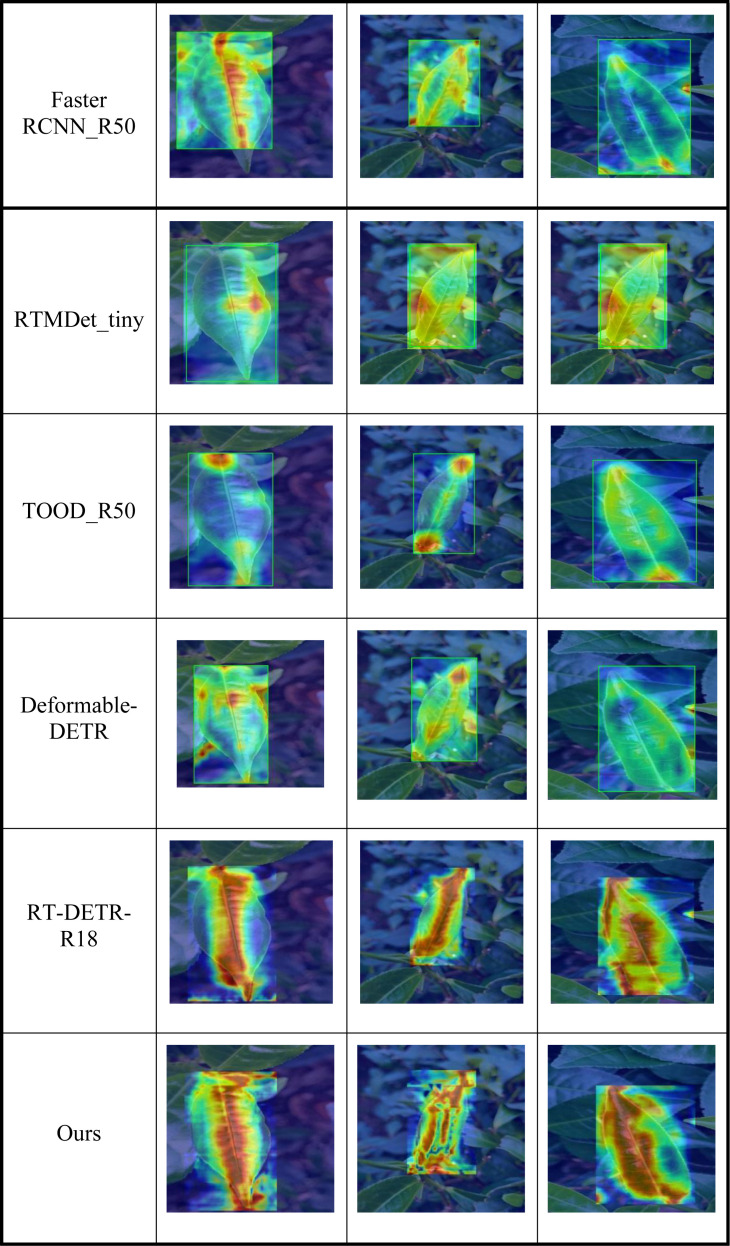
Detection and Grad-CAM++ visualization results of different models.

To further evaluate the practical detection capability and feature attention characteristics of different models, detection visualization results together with Grad-CAM++ heatmaps were analyzed under various tea leaf detection scenarios. The visualization results are shown in **Figure 10**.

From the detection results, the proposed model demonstrates more accurate localization performance and fewer false or missed detections under complex backgrounds, dense leaf distributions, and partial occlusion conditions. Compared with RT-DETR-R18, Faster R-CNN, RTMDet, TOOD, and Deformable-DETR, the proposed model generates more complete bounding boxes and maintains better detection consistency for overlapping and small-scale tea leaf targets.

From the perspective of feature attention, the Grad-CAM++ heatmaps generated by the proposed model exhibit highly concentrated activation regions on the main leaf body and key discriminative structures, including veins and lamina regions. In contrast, several comparative models produce relatively scattered or incomplete activations, with certain responses extending into background regions. This indicates that the proposed model possesses stronger capability in suppressing background interference and extracting representative target features.

Overall, both the detection visualization results and Grad-CAM++ analyses demonstrate that the proposed model achieves superior target localization accuracy and more effective feature representation under challenging tea leaf detection scenarios.

## 4 Discussion

Automatic tea-grade determination in open-field, complex tea-garden habitats faces several core challenges, including interference from unstructured backgrounds, unstable feature capture for small targets, and the inherent tension between lightweight design and detection accuracy. These issues also constitute key research topics in agricultural computer vision. Through targeted module-level optimization, Tea-DETR provides an effective technical route to address the above challenges, with the main advantages and innovations summarized as follows.

(1) Synergistic improvement of lightweightness and performance, alleviating the “lightweight–accuracy” trade-off.

Conventional RT-DETR suffers from a relatively large parameter size and insufficient inference efficiency when deployed on field edge devices. Naïve lightweighting often degrades feature extraction capability. By contrast, the proposed C2f-EViM-CGLU backbone preserves the multi-branch aggregation advantage of C2f, introduces EfficientViMBlock to enable long-range dependency modeling with approximately linear complexity, and integrates the ConvGLU gating mechanism to suppress irrelevant feature responses. As a result, Tea-DETR reduces parameters by 26.8% (to 14.65M) while improving mAP@0.5 to 82.0%. Compared with existing tea-leaf detection studies, the proposed method achieves a substantial performance gain purely through structural optimization, without relying on complex pretraining or multi-model ensembles, making it more suitable for real-time field detection.

(2) Robustness-oriented feature enhancement tailored to open-field characteristics, substantially improving grade discrimination.

In open tea gardens, weeds, illumination changes, and leaf overlaps frequently impair the capture of edge details and fine-grained textures of highly tender leaves (Grade T1). The proposed AIFI_EPGO_SHSA module addresses this by reducing attention redundancy and introducing dynamic Top-k sparsification, which concentrates the attention budget on key tokens relevant to tea-grade cues while suppressing background noise and ineffective interactions. This strategy improves overall accuracy to 92.2% and strengthens the model’s capability to distinguish subtle semantic differences among grades T1–T4. Unlike conventional approaches that indiscriminately enhance global features, the proposed design offers a transferable paradigm for fine-grained detection of other crop targets under complex backgrounds.

(3) Clear practical value and improved translational potential.

Accurate grading of Anji white tea is crucial for quality control and industrial value enhancement. Traditional manual grading is inefficient, subjective, and may damage tea leaves. Tea-DETR jointly achieves real-time inference and non-destructive inspection, is compatible with mobile high-resolution image acquisition, and can deliver accurate grading without complicated preprocessing. It can therefore be directly integrated into practical workflows such as tea-garden patrol inspection and harvest-time grading, providing enabling technology for digital transformation of the tea industry. In contrast to laboratory-based tea detection studies, the dataset in this work was collected under real open-field habitats, better reflecting real-world performance and strengthening translational value.

Despite these strengths, several limitations remain. First, the dataset covers only the core production area of Anji white tea in Zhejiang; samples from multiple varieties, regions, and extreme weather conditions are insufficient, which may limit generalization. Second, grade determination relies solely on visual cues, without incorporating multimodal information such as spectroscopy, and the discrimination of visually similar grades could be further improved. Third, although the model is lightweight, inference speed on low-compute edge devices still requires optimization and may not fully satisfy large-scale real-time field inspection.

Nevertheless, the current study is based on data collected from a single core production area, and variations in cultivation environments, illumination conditions, and leaf morphology across different regions may affect the generalization capability of the proposed model. Therefore, further validation on multi-region and multi-season datasets is still required before large-scale deployment in diverse field scenarios. Future work will focus on expanding the dataset to include diverse tea varieties, regions, and environmental conditions, and leveraging data augmentation to improve generalization. Multimodal fusion of vision and spectral features will also be explored to further enhance grading precision. In addition, edge deployment efficiency can be improved via model quantization and pruning to facilitate large-scale real-world applications. Finally, the proposed framework could be extended to other tea-garden tasks such as pest/disease detection and yield estimation, enabling an integrated intelligent monitoring system for tea plantations and providing broader support for smart agriculture.

## 5 Conclusion

This study addresses the technical bottlenecks of automatic tea-grade determination in open-field tea gardens with complex habitats by proposing and systematically validating an optimized model, Tea-DETR. The main conclusions are as follows. First, through coordinated optimization of the backbone network and feature enhancement modules, the proposed model effectively alleviates key challenges, including suppression of background interference, stable feature capture for small targets, and the trade-off between lightweight design and accuracy, thereby significantly improving the robustness and reliability of tea-grade determination under complex field conditions. Second, experiments demonstrate that Tea-DETR achieves a 26.8% reduction in parameters (down to 14.65M) while improving the accuracy and mAP@0.5 to 92.2% and 82.0%, respectively, indicating that it balances real-time inference performance with high grading precision and exhibits strong potential for practical engineering deployment. Third, this work provides reliable technical support for real-time, non-destructive automatic tea-grade assessment in field scenarios, and offers important practical value for promoting digital transformation of the tea industry and the deployment of smart-agriculture technologies. Finally, to address the limitations of this study, future work may further enhance generalization and scalability by expanding dataset coverage, integrating multimodal features, and optimizing edge-deployment strategies.

## Data Availability

The data analyzed in this study is subject to the following licenses/restrictions: Tea Grading. Requests to access these datasets should be directed to 63532718@qq.com.
